# Pathogenicity of two *COQ7* mutations and responses to 2,4‐dihydroxybenzoate bypass treatment

**DOI:** 10.1111/jcmm.13154

**Published:** 2017-04-13

**Authors:** Ying Wang, Christopher Smith, Jillian S. Parboosingh, Aneal Khan, Micheil Innes, Siegfried Hekimi

**Affiliations:** ^1^ Department of Biology McGill University Montréal Quebec Canada; ^2^ Department of Medical Genetics Alberta Children's Hospital University of Calgary Calgary Alberta Canada; ^3^ Alberta Children's Hospital Research Institute for Child and Maternal Health University of Calgary Calgary Alberta Canada; ^4^ Metabolic Diseases Clinic Alberta Children's Hospital University of Calgary Calgary Alberta Canada

**Keywords:** *COQ7*, coenzyme Q, ubiquinone, primary ubiquinone deficiency, mitochondrial dysfunction, 2,4‐dihydroxybenzoic acid

## Abstract

Primary ubiquinone (co‐enzyme Q) deficiency results in a wide range of clinical features due to mitochondrial dysfunction. Here, we analyse and characterize two mutations in the ubiquinone biosynthetic gene *COQ7*. One mutation from the only previously identified patient (V141E), and one (L111P) from a 6‐year‐old girl who presents with spasticity and bilateral sensorineural hearing loss. We used patient fibroblast cell lines and a heterologous expression system to show that both mutations lead to loss of protein stability and decreased levels of ubiquinone that correlate with the severity of mitochondrial dysfunction. The severity of L111P is enhanced by the particular COQ7 polymorphism (T103M) that the patient carries, but not by a mitochondrial DNA mutation (A1555G) that is also present in the patient and that has been linked to aminoglycoside‐dependent hearing loss. We analysed treatment with the unnatural biosynthesis precursor 2,4‐dihydroxybenzoate (DHB), which can restore ubiquinone synthesis in cells completely lacking the enzymatic activity of COQ7. We find that the treatment is not beneficial for every *COQ7* mutation and its outcome depends on the extent of enzyme activity loss.

## Introduction

Ubiquinone (UQ; Co‐enzyme Q, CoQ) is a redox‐active lipid essential for multiple cellular functions. In particular, it is a pivotal component of the mitochondrial respiratory chain (RC) where it functions as an electron carrier shuttling electrons from complex I, complex II and a variety of other dehydrogenases, to complex III, from which electrons continue to flow through the remainder of the RC 1. Other functions of UQ include a dual role in both relative oxygen species (ROS) production and protection from ROS, as well as participation in trans‐plasma membrane electron transport 2. A UQ molecule is composed of a benzoquinone ring and an isoprenoid tail of different length depending on the organism. In humans, the side chain of UQ contains 10 isoprene units (UQ_10_), while in mice the predominant UQ species is UQ_9_. The quinone head is the functional group, whereas the isoprenoid tail primarily serves to anchor UQ to the membrane, as UQ species with different tail lengths can functionally substitute for each other in many situations 3, 4. UQ_10_ supplementation is well tolerated but the highly lipophilic nature of UQ due to the presence of the long isoprenoid side chain allows only for poor incorporation into intracellular membranes (at least *in vivo*). In mice, it was shown that dietary UQ_10_ is not taken up by most tissues, except by the liver, ovary and brown adipose tissue 5, 6, 7.

Endogenous UQ biosynthesis in mammals requires at least 10 genes (*Pdss1*,* Pdss2*,* COQ2* to *COQ9*), and is carried out in the inner mitochondrial membrane. Primary UQ deficiency (MIM#607426) due to genetic defects in the UQ biosynthetic pathway represents an important group of inherited mitochondrial diseases. It is often associated with multiple organ dysfunction, significant morbidity and poor patient outcomes. Most patients are juvenile onset and present with severe mitochondrial disease phenotypes 8, 9, 10, 11, 12, 13, 14, 15, 16, 17. Supplementation with exogenous UQ is currently the only available treatment for UQ deficiency. However, the effect of the treatment is often minimal, and only some patients respond to it 18.

Mutations in eight of the 10 genes currently known to be necessary for UQ biosynthesis have been reported 8, 9, 10, 11, 12, 13, 14, 15, 16, 17, 19, 20. Recently, Freyer *et al*. reported the first UQ deficiency patient carrying a missense mutation in the *COQ7* gene 19. *COQ7* [GenBank: NM_016138.4], also known as *Mclk1* in mouse and *clk‐1* in *C. elegans*, is responsible for the penultimate step of UQ biosynthesis, converting 6‐demethoxyubiquinone (DMQ) to 6‐hydroxyubiquinone which is then turned into UQ by the methylase COQ3. Accordingly, animal cells with reduced COQ7 activity have low levels of UQ and show accumulation of the biosynthetic precursor DMQ 4, 5, 21, 22, 23, 24, 25. It has not yet been clearly established whether the accumulated DMQ has phenotypic effects in these mutants 4, 5, 25.

Studies of yeast and mouse mutants have shown that UQ biosynthesis can be restored in cells and animals that lack the COQ7 orthologue by treatment with 2,4‐dihydroxybenzoic acid (DHB) 5, 26. DHB differs from the native UQ biosynthetic precursor 4‐hydroxybenzoic acid (4‐HB) by already having a hydroxyl group at carbon C6, which is normally hydroxylated by COQ7, allowing to bypass the need for COQ7 in UQ synthesis 26. The skin fibroblasts of the previously reported *COQ7* patient also appear to respond to DHB with an increase in UQ_10_ levels 19. However, it is important to note that although UQ can be made from DHB without COQ7, DHB also inhibits the native UQ biosynthetic pathway, probably due to competition for access to the enzymes upstream of the COQ7‐catalysed enzymatic step.

In this study, we report a patient that carries a novel homozygous mutation in *COQ7* (L111P) as well as a 1555A>G mutation in mtDNA that has been associated with aminoglycoside(AG)‐induced, non‐syndromic deafness 27. The main symptoms of the patient we report here are spasticity and hearing loss. Using patient fibroblast cell lines and a heterologous expression system, we first show that both *COQ7* mutations (the mutation first described here as well as the mutation from the only previously identified patient 19) lead to a drastic decrease of protein stability and consequently a reduction in ubiquinone levels. We also demonstrate different degrees of severity for the two mutations and how the severity of the novel mutation (L111P) depends on another polymorphism in the *COQ7* gene but not on the presence of the 1555A>G mtDNA mutation. Furthermore, we reveal how the two patients' mutations respond differently to DHB and UQ_10_ treatment, providing insight that will help to devise treatment options for *COQ7* patients.

## Materials and methods

### Patient profile

The patient was the first child of consanguineous healthy parents who are first cousins once removed. She was born at 37 weeks of gestational age, after a pregnancy associated with gestational diabetes. She was healthy in the immediate newborn period and early infancy, being discharged from hospital after 48 hrs. While her early developmental milestones were normal, concerns became apparent with her motor milestones in the second year of life. Her developmental delay became particularly noted, after a urinary tract infection at 14 months of age. It required antibiotic treatment for which ceftriaxone was given. There was also a question of language delay, initially attributed to the child being exposed to two languages at home.

By age 2, she was not yet walking and upon assessment was found to have increased tone in the lower extremities. Her tone in the upper extremities was normal and her fine motor skills appeared age appropriate. She was trialled on carbidopa/levodopa for 12 weeks and later baclofen and botox injections, all with limited to no effect. At the age of 5 years, she was diagnosed with bilateral low frequency, rising to normal, sensorineural hearing loss for which she wears hearing aids.

The patient is now 6 years of age. On examination, she has normal height, weight and head circumference and is non‐dysmorphic. She has good head control and mild truncal hypotonia. The examination is mostly notable for increased tone and spasticity in the lower extremities, with 3+ reflexes bilaterally and clonus particularly on the right side. The Babinski response was positive on both sides. There was generalized muscle wasting, more prominent in the legs but also affecting the temporalis muscles despite lack of muscle weakness or spasticity in the head and neck. Eye movements, facial expression, chest examination and abdominal examination did not reveal any abnormalities.

At age 6 years 2 months, the patient had formal psychoeducational testing which highlighted some concerns regarding inattention as well as early academic skills in the low‐average range. She never learned to walk independently and had poor balance with the ability to walk only with support at 18 months of age. She had been wheelchair bound since 3 years of age. Given progressive worsening of her mobility, at 5 years she had derotational surgery of hips, which facilitated her positioning and has allowed her to walk with a walker.

Initial investigations included serial normal MRI scans of the brain and spine, normal renal ultrasound, normal urine metabolic screen, normal plasma and urine amino acids, normal urine organic acids, and normal plasma acyl‐carnitine profile and carnitine levels. Blood creatine kinase and lactate levels were within normal limits at rest, but the concentration of lactate in the cerebrospinal fluid (CSF) was elevated at 2.8 mmol/l (reference range 1.1–2.4). CSF glucose is normal at 2.9 mmol/l (reference range 2.2–3.9), and total protein was slightly decreased at 0.13 g/l (reference range 0.15–0.45). The levels of amino acids, neurotransmitter metabolites and 5‐methyltetrahydrofolate were found not to be affected in the CSF.

Both of her parents are healthy. Family history reveals no individuals similarly affected with spasticity or abnormal motor development. There is, however, a strong family history of retinitis pigmentosa affecting, among others, the paternal grandmother and the maternal great‐grandmother. Given a family history of retinitis pigmentosa, she has had formal ophthalmology assessments which have been normal.

Due to her clinical presentation with increased spasticity in the lower limbs, an uneventful delivery history, normal MRI scan and parental consanguinity, a tentative clinical diagnosis of hereditary spastic paraplegia (HSP) was applied. Due to the high genetic heterogeneity of HSP, whole‐exome sequencing was pursued on a research basis. The parents of the patient provided informed consent. The patient recently started supplementation of UQ_10_ at 11.4 mg/kg bid and alpha‐lipoic acid 100 mg po daily. At 3 months' follow‐up, she has not had deterioration or worsening spasticity, but no obvious improvement could be seen.

### Whole‐exome analysis

Whole‐exome sequencing was performed at the Alberta Children's Hospital Research Institute Genomics and Bioinformatics Facility. Genomic DNA from the affected individual underwent exome enrichment with the Agilent SureSelect V5 Kit (Agilent Technologies, Santa Clara, CA, USA) prior to sequencing with a 5500xl SOLiD sequencer (Thermo Fisher Scientific, Waltham, USA). Due to the presence of parental consanguinity, we searched for homozygous recessive variants of putative functional impact including missense, nonsense, splice site mutations and indels (insertions and deletions). A novel, homozygous missense mutation in the gene *COQ7* for which both parents were heterozygous was identified in the proband. For mitochondrial DNA (mtDNA) sequencing, long‐range PCR products from the DNA extracted from muscle biopsy were used as the template. mtDNA sequencing and Southern Blot analysis were performed by Alberta Health Services. It revealed one potentially pathogenic mutation in her mtDNA: the A1555G base substitution in the 12S rRNA gene.

### Confirmation of mutation detection and RFLP analysis

RNA was extracted from patients' skin fibroblasts by use of TRIzol reagent (Thermo Fisher Scientific, Waltham, MA, USA) and reverse‐transcribed into cDNA using a Quantitect reverse transcription kit (Qiagen, Valencia, CA, USA), according to the manufacturers' protocols. The full‐length coding sequence of COQ7 was then amplified by PCR using AccuPrime Taq DNA Polymerase High Fidelity (Thermo Fisher Scientific) with the primer pair: forward, 5′‐ GTCCGAGCCAAGGGCACTAT‐3′; and 5′‐GACAGGCAAAACTGGACACAC‐3′. PCR products were directly used for sequencing (McGill University and Génome Québec Innovation Centre).

To confirm the presence or absence of the m.1555A>G mutation, a part of 12S rRNA gene harbouring the mutation region was amplified by PCR from genomic DNA using AccuPrime Taq DNA Polymerase High Fidelity (Thermo Fisher Scientific). The following primers, which cover nucleotides 1323 ‐ 1965 of the mtDNA, were used: forward 5′‐AAAGACGTTAGGTCAAGGTG‐3′ and reverse 5′‐GCTACATAGACGGGTGTGCTC‐3′. The 643‐bp PCR product was subjected to sequencing using the same primers as well as restriction fragment length polymorphism (RFLP) analysis using BsmAI (New England Biolabs). The digestion of PCR products from samples without the 1555A>G mutation produces two fragments of 409 and 234 bp due to the recognition of the restriction site of BsmAI. The samples with the 1555A>G mutation only yields a single fragment of 643 bp, because the presence of the mutation abolishes the BsmAI recognition site.

### Reagents and cell culture

All chemicals were purchased from Sigma‐Aldrich (Oakville, ON, Canada), unless otherwise specified. Dulbecco's modified Eagle's medium (DMEM), trypsin and antibiotic–antimycotic solution were purchased from Wisent Bioproducts. Other cell culture reagents were obtained from Thermo Fisher Scientific. Skin fibroblasts were derived from skin biopsy following standard procedures. Normal control fibroblasts were kindly provided by Dr Eric Shoubridge and Dr Hana Antonicka (McGill University, Montreal, QC, Canada). All cells were routinely maintained in DMEM supplemented with 10% foetal bovine serum and 1% antibiotic–antimycotic solution at 37°C and 5% CO_2_. Cells with passage numbers between 8 and 20 were used in all experiments. For stimulation of oxidative phosphorylation, medium with no glucose but containing galactose (10 mM) was used. For treatment with DHB or UQ_10_, unless otherwise specified, the chemicals were added into culture media for 1 week before cells were used for analyses.

### Retroviral vectors and gene transfer

The coding region for COQ7 was generated by PCR amplification from a full‐length human COQ7 cDNA (HG12195‐G; Sino Biological Inc., Beijing, China) and cloned into the BamH1 and EcoR1 sites of pBabe‐hygro (obtained from Addgene). The resulting retroviral expressing vector is named pBabe‐hCOQ7‐hygro. The sequences of the primers used are the following: forward primer, 5′‐CGATGAGGATCCATGAGTTGCGCCGGGGC‐3′; reverse primer, 5′‐ TGTGCTGAATTCTTATAATCTTTCTGATAAATATATCGCCACTCTGC‐3′.

To introduce a particular mutation(s) into pBabe‐hCOQ7‐hygro, QuikChange^®^ II site‐directed mutagenesis kit (Agilent Technologies) was used accordingly to the manufacturer's instructions. The correct insertion of the mutations was verified by plasmid sequencing.

To generate *Mclk1* KO MEFs that stably express the wild‐type or a mutant *COQ7*, Phoenix packaging cells were transfected with retroviral plasmids using Lipofectamine 2000 reagent (Thermo Fisher Scientific). At 48 hrs after transfection, the viral supernatant recovered from cultures of transfected Phoenix cells was filtered through a 0.45‐μm filter and added to the MEF cultures with polybrene at 8 μg/ml. The cells were then cultured in the presence of hygromycin B (200 μg/ml) for 2 days to select for infected cells.

### Quantitation of ubiquinone

Quinone extraction and quantitation by high‐performance liquid chromatography (HPLC) were carried out as previously described 4, 28, 29. Briefly, cells were lysed in a radioimmunoprecipitation buffer (Tris–HCl, pH 7.5, 1% NP‐40, 0.5% deoxycholate, 10 mM EDTA, 150 mM NaCl) and extracted with a mixture of ethanol and hexane (2:5, v/v) for 2 min. by vigorously vortexing. After centrifugation, the upper organic layer was transferred to a new tube and hexane was evaporated by drying in a SpeedVac concentrator. The left residual was finally redissolved in ethanol before injection into HPLC (a Beckman Coulter System Gold HPLC system with 32 Karat software). During the HPLC, the samples were separated on a reverse‐phase C18 column (25.0 × 0.46 cm, 5 μm, Highchrom), eluted with mixture of methanol and ethanol (7:3, v/v) at 1.8 ml/min and detected at 275 nm. Peak identities were established by comparison with the profile obtained from ubiquinone‐10 standard (Sigma‐Aldrich). Protein content in the quinone extracts was determined by the BCA assay (Thermo Fisher Scientific) and used to normalize quinone levels.

### Measurement of oxygen consumption by Seahorse XF24 Extracellular Flux Analyzer

Oxygen consumption of intact cells was determined using a Seahorse Bioscience XF24 Extracellular Flux Analyzer following the manufacturer's instructions. Briefly, cells at a density of ~ 30,000 per well were seeded in XF v7 24‐well plates for 24 hrs. Prior to the measurements, cell medium was replaced with Seahorse XF base medium supplemented with 10 mM galactose, 2 mM Glutamax and 1 mM sodium pyruvate and allowed to equilibrate for 1 hr at 37°C. Oxygen consumption was measured under basal conditions, and in the presence of oligomycin (1 μg/ml), FCCP (0.8 μM), and a combination of rotenone (1 μM) and antimycin A (5 μM) was used to assess the basal and maximal mitochondrial respiration rates. Four baseline measurements were recorded before the consecutive addition of the above‐mentioned compounds, and three response measurements were taken after the addition of each compound. To normalize respiration rates to protein content, cells were lysed with the CelLytic M Lysis reagent (Sigma‐Aldrich) and protein concentrations were measured using the Bradford protein assay (Bio‐Rad, Mississauga, ON, Canada). Basal respiration rate was calculated as oxygen consumption rate (OCR) before oligomycin injection minus OCR after rotenone plus antimycin A injection. Maximal was determined as the OCR after FCCP minus non‐mitochondrial OCR after injection of the ETC. inhibitors.

### Muscle biopsy

An open biopsy of the right vastus lateralis was performed using a standard protocol 30. Frozen samples were processed for mitochondrial protein separation by Bis‐Tris polyacrylamide gel electrophoresis and assaying of the in‐gel activities of the RC complexes (I‐V) (Medical Neurogenetics, Atlanta, GA, USA). In addition, H&E, Gomori's trichrome and oil red O staining were performed to search for ragged red fibres and detection of abnormal lipid deposit. Stains for NADH, succinic dehydrogenase, cytochrome oxidase or ATPase (at pH 4.3, 4.7, and 10.4) were performed in order to identify fibres with RC abnormalities. Electron microscopy was performed by the Calgary Laboratory Services (CLS) Pathology Laboratory according to standard techniques.

### Immunoblotting

Crude mitochondrial extracts corresponding to 40 μg of protein were resolved on 12% SDS‐PAGE gels and transferred to nitrocellulose membranes (Bio‐Rad). The primary antibodies used were rabbit anti‐MCLK1/COQ7 (generated in our own laboratory) and anti‐VDAC/Porin (Cell Signaling Technology, Danvers, MA, USA) at 1:1000 dilution each. The membrane was incubated with the primary antibodies overnight at 4°C, followed by secondary HRP‐conjugated anti‐rabbit IgG (Cell Signaling Technology) for 1 hr at room temperature. After three washes, the membrane was developed with ECL Western blotting detection reagent (GE Healthcare, Mississauga, ON, Canada) and visualized by exposure to X‐ray film.

### Statistical analysis

All quantitative results are expressed as mean ± standard error of the mean (S.E.M.) or ± the standard deviation (S.D.) as indicated. Depending on experimental circumstances, appropriate analysis of variance (anova) or *t* tests were performed with graphpad prism version 6.0 (GraphPad Software, Inc. La Jolla, CA, USA).

### URLs

Exome Aggregation Consortium (ExAC) database: http://exac.broadinstitute.org/, PolyPhen‐2 software: http://genetics.bwh.harvard.edu/pph2/; SIFT software: http://sift.jcvi.org/; and MutationTaster: http://www.mutationtaster.org/ for prediction of mutation effects, The 1000 Genomes Browser: http://browser.1000genomes.org/index.html, MITOMAP: http://www.mitomap.org/MITOMAP, Rare Diseases Models and Mechanisms Network, http://www.rare-diseases-catalyst-network.ca/index.php.

## Results

### Identification of a novel COQ7 mutation and the classical mitochondrial DNA mutation A1555G in the same patient

**Figure 1:**
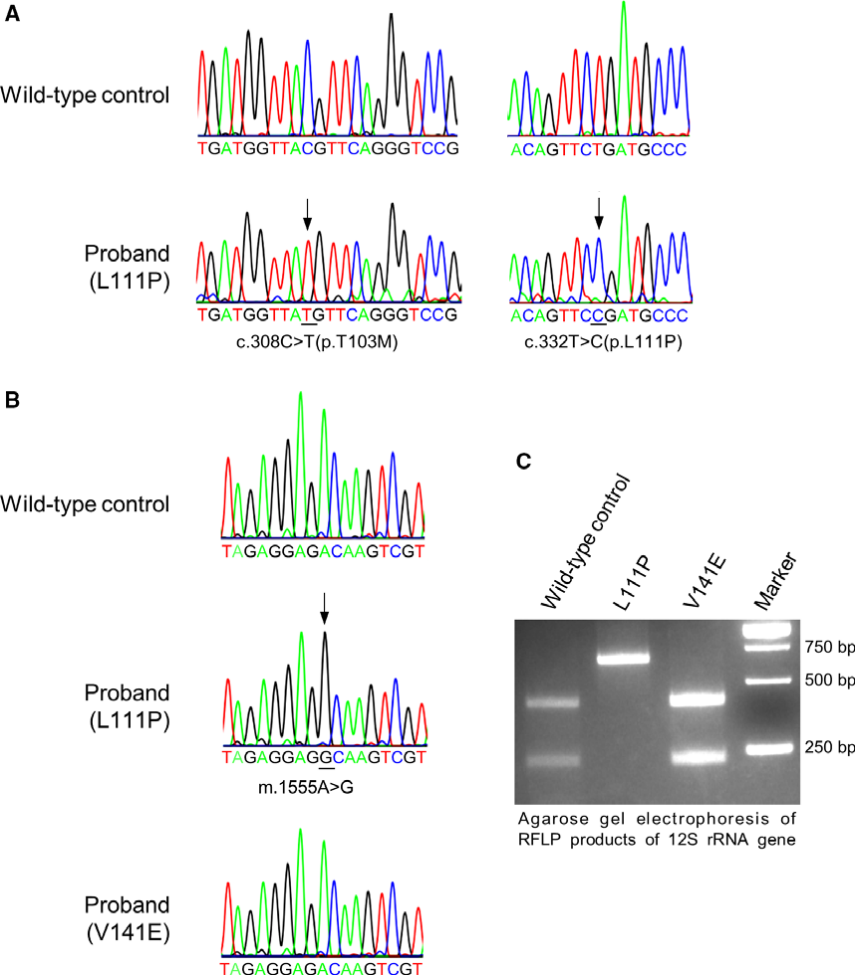
*COQ7* gene variants and a mitochondrial DNA mutation identified in the patient under study. (**A**) *COQ7* gene variants. Sequencing chromatograms are shown for the c.308C>T and c.332T>C variants detected in *COQ7* in the proband reported in this study, with the wild‐type sequence at the top. The chromatograms also show that the patient is homozygous for both the c.308C>T [p. Thr103Met] polymorphism and the c.332T>C [p.Leu111Pro] mutation. (**B**) Chromatograms showing a partial sequence of the mitochondria 12S rRNA gene in a normal control, the L111P patient reported in this study or the previously reported *COQ7* patient carrying the V141E mutation. Both patients and the normal control used in this study carry the m.1438A>G polymorphism. The L111P patient, in addition, carries the m.1555A>G mutation. Black arrows denote altered nucleotides. (**C**) RFLP of mitochondrial DNA. Shown is an agarose gel electrophoresis of PCR products from the mitochondrial 12S rRNA gene digested by BsmAI. Wild‐type control: skin fibroblasts from a healthy unrelated individual; L111P: the proband reported in this study; V141E: *COQ7* patient previously reported in 19. The PCR product (643 bp) without the m.1555A>G mutation was cleaved in two fragments of 409 and 234 bp, which was the case for the wild‐type control and the V141E patient. The PCR product from the L111P patient's 12S rRNA gene was not cut by BsmAI, as the m.1555A>G mutation abolishes the BsmAI restriction site. Moreover, only a single band corresponding to the mutated fragment was seen in the sample from the L111P patient. This, together with the sequencing chromatogram, demonstrates homoplasmy for the m.1555A>G mutation.

In order to identify the genetic cause of the patient's clinical findings, we first performed whole‐exome sequencing on the patient's and her parents' genomic DNA. The search revealed two mutations in the *COQ7* gene of the patient: NM_016138:c.308C>T [p. Thr103Met] and c.332T>C [p.Leu111Pro]. Sanger sequencing showed that both parents were heterozygous for both variants and confirmed that the variants was homozygous in the affected proband. These two mutations were further confirmed by sequencing the whole coding region of *COQ7* cDNA from the patient's fibroblasts (Fig. 1A). We queried the Exome Aggregation Consortium (ExAC) database which contains allele frequency information generated from exome sequencing data from 61,486 unrelated individuals. It was found that the c.308C>T [p. Thr103Met] variant (simply referred to as T103M below) has an allele frequency of 0.627 with 24314 homozygotes. It is also listed in the single nucleotide variants of the 1000 genomes database with a reported minor allele frequency of 0.425. These observations suggest that the variant is likely a benign polymorphism. In contrast, the variant c.332T>C [p.Leu111Pro] (referred to as L111P below) is exceptionally rare and most likely deleterious, as in ExAC it has a frequency of 0.00001726 in 2/115866 chromosomes and with no homozygotes. In addition, SIFT, PolyPhen‐2 and MutationTaster all predict this variant to be pathogenic (SIFT score = 0.00, PolyPhen‐2 score = 1.00) 32, 33, 34. The 111th amino acid residue is at a highly conserved site with a GERP conservation score of 5.91, but is not considered to be part of the iron‐binding motif 34.

We also identified a heterozygous stop‐gain mutation in the previously reported recessive retinitis pigmentosa gene *C2ORF71* 35, likely explaining the family history of that condition (particularly given that the obligate carrier father also carried this variant). Sanger sequencing of the mtDNA of the patient's muscle and Southern blot analysis did not identify any known mutation leading to mitochondrial disease. However, there was the discovery of a 1555A>G change in the MT‐RNR1 (12S rRNA) gene (Fig. 1B and C) as well as the 1438A>G polymorphism (data not shown). The mitochondrial 1555A>G mutation has been reported worldwide in diverse populations, with a frequency of ~ 0.17%. It was suggested to be an important cause of AG‐induced non‐syndromic hearing loss 27, 36, 37, 38. The mutation has been documented also in hearing‐impaired individuals who were not exposed to AGs 38, 39. It is not yet clear whether, in fact, and why exactly the mutation renders affected individuals a high risk of hearing loss 40, 41. For the 1438A>G polymorphism in the 12S rRNA gene, the Mitomap database reports its population prevalence as high as 94%. We also detected it in the normal control we used in this study (data not shown).

### Different degrees of pathogenicity of distinct COQ7 mutations

**Figure 2:**
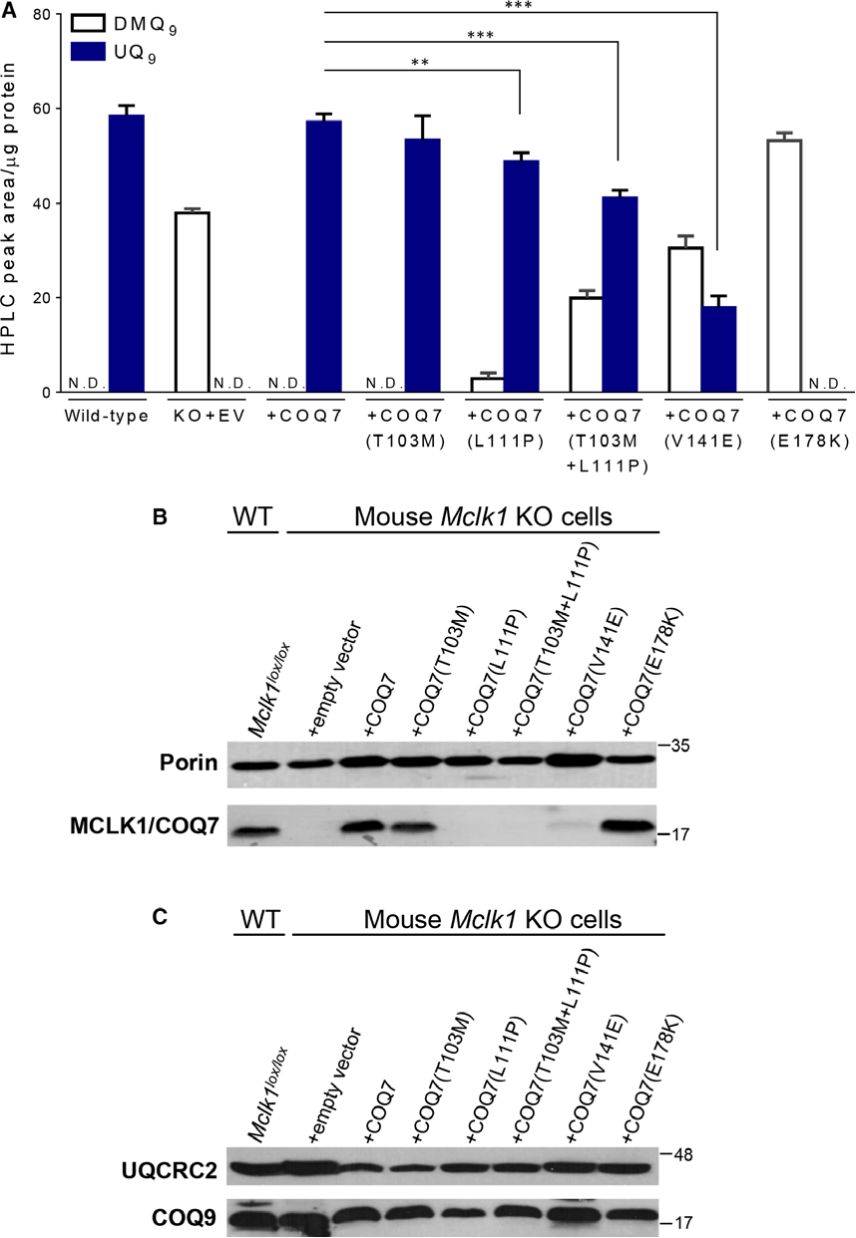
The pathogenicity evaluation of the patients' mutations in the *COQ7* gene (**A**) UQ levels in the *Mclk1/Coq7* KO MEF expressing or not wild‐type or mutated human *COQ7*. The c.308C>T [p.Thr103Met] (T103M) polymorphism had no significant effect on UQ levels, while the KO cells carrying the c.332T>C [p.Leu111Pro] mutation (L111P) in the transduced *COQ7* showed a mild reduction in UQ_10_ levels and a small amount of DMQ_10_. L111P, when present together with T103M, reduced the level of UQ to a greater degree than by itself. The c.422T>A [p.Val141Glu] mutation (V141E) resulted in a more severe loss of UQ_10_ and a larger accumulation of DMQ_10_ compared with the L111P mutation. No UQ_10_ was detected in the KO cells expressing *COQ7* with the c.532G>A mutation which results in a Glu178Lys (E178K) change corresponding to the *C. elegans* mutant *clk‐1(2519)*. Quinone levels are expressed as HPLC‐UV peak area normalized to protein content. N.D.: Not detectable. EV: empty vector. Data are mean ± S.E.M. (*n* = 4–6). **P < 0.01, ***P < 0.001 (Student's *t*‐test). (**B**) Western blot analysis of COQ7 levels. The first lane shows MCLK1 in wild‐type control MEFs (mock‐infected *Mclk1* ^*lox/lox*^ cells), while the other lanes show the human COQ7 band detected in the *Mclk1* KO cells expressing or not wild‐type or mutated COQ7. Extremely low levels of COQ7 were expressed in the KO cells carrying the mutated cDNA of *COQ7* that contain the patients' mutations (*i.e*. L111P, T103M+L111P or V141E). The polymorphism T103M did not significantly affect the COQ7 protein level. Increased COQ7 expression was observed in the KO cells expressing the mutant COQ7 protein with a E178K change. (**C**) Western blot of COQ9. No significant decrease in mouse COQ9 levels was observed in the cells that have nearly undetectable levels of MCLK1 or COQ7. Western blot was performed on mitochondrial protein extracts and mitochondrial Porin/VDAC and ubiquinol‐cytochrome C reductase core protein II (UQCRC2) were used as loading controls. The migration positions of protein size markers (in kD) are shown on the right. Two Western blots shown were performed on different preparations of mitochondrial proteins. Uncropped Western blot images are shown in Figure S5.

In 2015, Freyer *et al*. described a patient carrying a homozygous c.422T>A [p.Val141Glu] mutation in the *COQ7* gene (referred to as V141E below)^19^. This patient is seriously ill and has multiple organ dysfunction. A severe decrease in UQ_10_ levels was observed in mitochondria from the patient's skeletal muscles as well as in cultured skin fibroblasts. However, whether the patient's mutation was in fact pathogenic has not been clearly established. To test the pathogenicity of the mutations in both the L111P and V141E patients, we used a heterologous expression system where mutant mouse cells that are deficient for MCLK1 (the orthologue of COQ7) express the human *COQ7* gene. *Mclk1* KO mouse embryonic fibroblasts (MEFs) were generated as previously described 4. They are unable to produce UQ_9_, but instead accumulate the biosynthetic precursor DMQ_9_. We introduced the full coding sequence of the human *COQ7* gene, with or without mutations, into *Mclk1* KO cells, and determined the recovery of UQ biosynthetic activity by measuring UQ levels. As shown in Figure 2A, stable expression of WT *COQ7* cDNA rescued UQ biosynthesis completely, whereas expression of *COQ7* carrying the mutations under study resulted in only a partial restoration of UQ levels. As mentioned above, the T103M base change is a polymorphism. This is confirmed by the fact that, by itself, it has no significant effect on UQ_9_ levels. In contrast, the L111P mutation reduced UQ production, demonstrating its deleterious effect on protein function. Interestingly, the combination of T103M and L111P produced a much greater decrease in UQ levels than L111P alone. Thus, it is not clear whether the effect of L111P in another background, where the amino acid at position 103 is threonine, would lead to clinical symptoms. In the cells that express COQ7 with the V141E mutation, a much more severe loss of UQ was detected. This is consistent with the fact that the clinical phenotype of the V141E patient is more severe than that of the patient first described here. Lastly, as an additional control, we introduced the c.532G>A[p.Glu178Lys] mutation into *COQ7*. This change, referred to as E178K below, corresponds to the *clk‐1(e2519)* mutation in *C*. *elegans* 24. Like in the nematode *clk‐1* mutant, no detectable UQ was produced in *Mclk1* KO cells carrying the E178K mutation. We also introduced each mutation into the mouse *Mclk1* gene and expressed them in the same system. The effects were comparable to those observed with the human gene (Fig. S1). Therefore, none of the effects is species specific.

### Drastically reduced levels of mutant COQ7 proteins

We tested the level of expression of the different mutated forms of COQ7 by Western blot analysis. Both the L111P and V141E mutations are predicted to give rise to full‐length COQ7 proteins with amino acid substitutions. To detect COQ7 in mitochondrial protein extracts, we used our anti‐MCLK1 antibody which was raised against a part of MCLK1 and recognizes both the mouse and the human forms of the protein 42. Expression of the wild‐type human COQ7 in *Mclk1* KO cells was similar to that of MCLK1 in control cells (*Mclk1* ^*lox/lox*^ cells infected with a virus that does not express the Cre recombinase) (Fig. 2B). The expression of COQ7 with methionine at position 103 (T103M polymorphism) was not different from that of the wild‐type control (with Thr103), indicating that the proteins are equally stable, consistent with their equal distribution in the population (Fig. 2B). In contrast, the expression of the mutant proteins carrying either of the two pathogenic mutations (L111P or V141E) dramatically lowered protein levels, which were almost equally undetectable for L111P whether or not the polymorphic site was Thr103 or Met103 (Fig. 2B). From this, we speculate that both L111P and V141E mutations decrease protein stability and thus the steady‐state COQ7 level and enzymatic activity. However, due to the limited sensitivity of Western blot assays, we cannot conclude that it is the difference in the severity of loss of protein expression that is responsible for the difference in enzyme activity. Interestingly, contrary to the patients' mutations, the E178K change, which affects the iron‐binding site of the protein, did not result in any reduction in COQ7 levels. Instead, similar to what was observed in the corresponding nematode mutant, the mutation produced high amounts of a non‐functional COQ7 protein 3. We also performed Western blotting analysis on the skin fibroblasts derived from the patients along with a normal control; unfortunately, the level of COQ7 expression in these cells was too low to be observed by immunoblotting. Finally, considering that decreasing the rate of general protein degradation may help stabilize the proteins with L111P or V141E mutations, we treated the KO cells carrying the mutations with MG132 (carbobenzoxy‐Leu‐Leu‐leucinal), a well‐recognized proteasome inhibitor 43. We did not however detect a significant gain of UQ levels following the treatment (data not shown).

### Uncoupling of loss of MCLK1/COQ7 from degradation of COQ9

In budding yeast, loss of any one COQ enzyme results in degradation of other UQ biosynthetic proteins, suggesting a multiprotein complex is involved in UQ biosynthesis 44. Whether a similar complex exists in animals has not yet been resolved. Among other Coq proteins, Coq7p and Coq9p are constituents of the yeast UQ biosynthetic complex 45. In mice, loss of *Coq9* was shown to induce a significant reduction in MCLK1/COQ7 protein levels 23, 46. However, it is not yet clear whether it is a result of disassembly of a putative UQ biosynthetic complex. We asked whether the cells with decreased expression of MCLK1/COQ7 show a reduction in COQ9 levels, which would be expected if a UQ biosynthesis protein complex also exists in animal cells. By Western blot, we found that the level of COQ9 protein was not affected in *Mclk1* KO cells or in the KO cells carrying the patients' mutations, all of which showed no or minimal COQ7 expression (Fig. 2C). This and the fact that *clk‐1* in *C. elegans* and mouse *Mclk1* KO mutants accumulate DMQ_9_, an intermediate for which all biosynthetic steps upstream of CLK‐1/MCLK1/COQ7 are required, indicate that UQ biosynthesis does not occur *via* a multi‐subunit COQ polypeptide complex in animal cells, or, if it does, that the complex is not disassembled in the absence of CLK‐1/MCLK1/COQ7.

### No increase in UQ levels by DHB treatment in L111P *COQ7* cells

**Figure 3:**
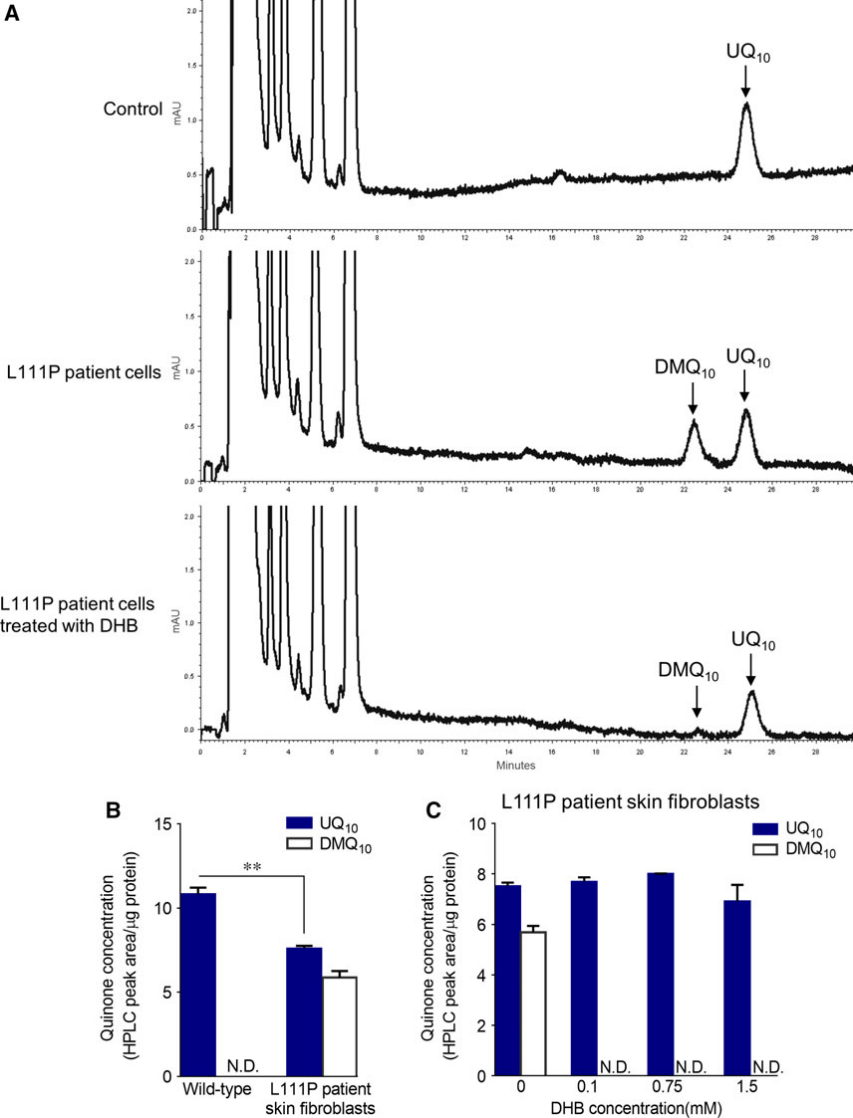
UQ content in the skin fibroblasts from the L111P *COQ7* patient. (**A**) HPLC chromatograms of quinone extracts from human skin fibroblasts. The L111P patient's cells showed ~ 30% decrease in UQ_10_ level and accumulation of the biosynthetic precursor DMQ_10_. Treatment of the patient cells with 1.5 mM DHB abolished the accumulation of DMQ_10_, but did not significantly increase UQ_10_ level. Cells with the same amount of protein (2.5 mg) were used for quinone extraction and HPLC quantitation. See Figure S2 for further verification of the peak identities. (**B**) Quinone levels expressed as HPLC‐UV peak area normalized to protein content. **P < 0.01 (Student's *t*‐test). (**C**) Quinone levels in the L111P skin fibroblasts after treatment with different doses of DHB for 7 days. N.D.: Not detected. Data represent the mean ± S.E.M. of 2–3 independent samples. Two‐way anova followed by Dunnett's post‐test revealed no statistically significant group difference in UQ_10_ concentrations.

**Figure 4:**
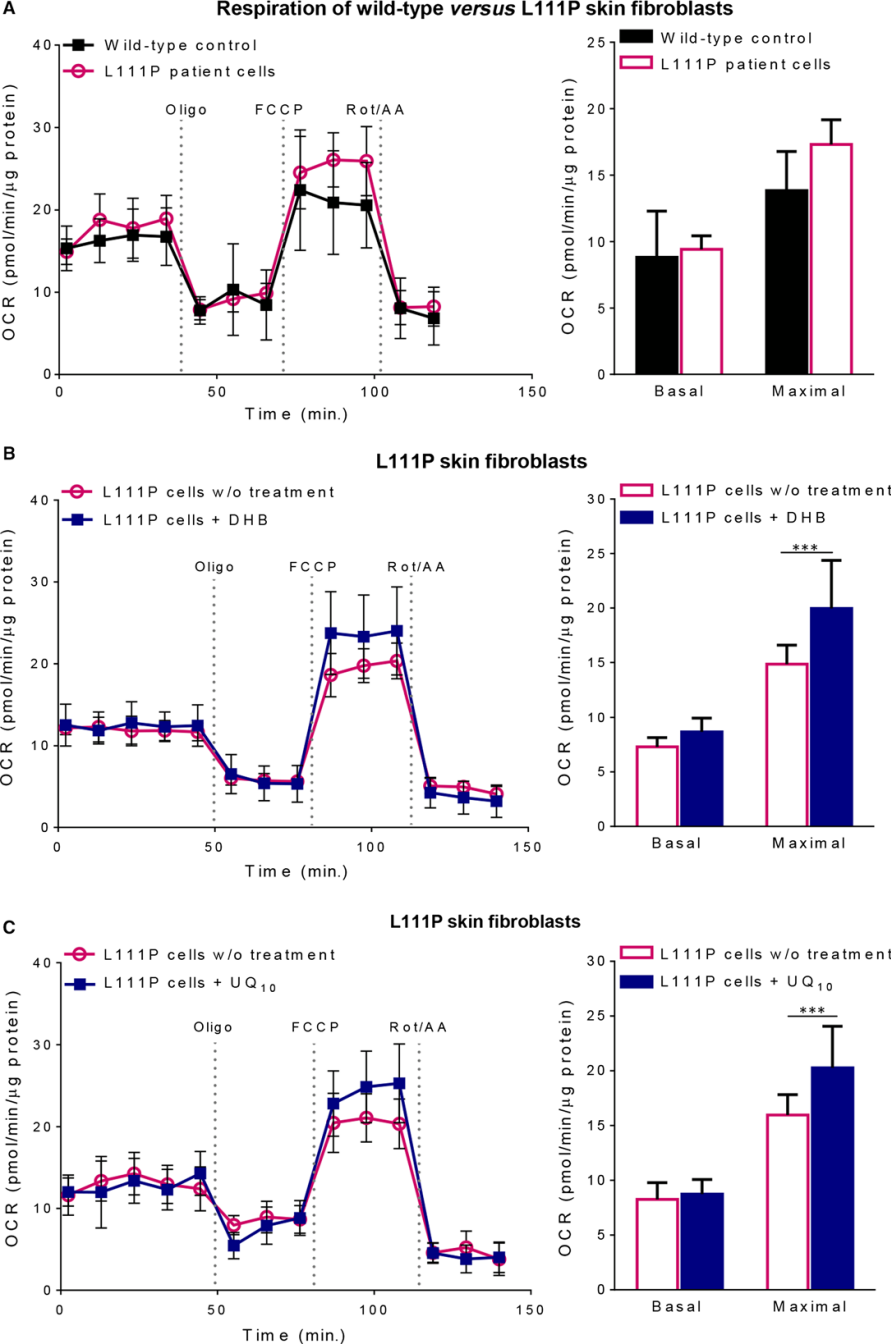
Oxygen consumption rates in L111P skin fibroblasts. After baseline OCR was established, ATP synthase inhibitor oligomycin (oligo), oxidative phosphorylation uncoupler carbonyl cyanide p‐[trifluoromethoxy]‐phenyl‐hydrazone (FCCP) and a mixture of complex I inhibitor rotenone (Rot) plus complex III inhibitor antimycin A (AA) were injected sequentially into each well to derive mitochondrial respiratory parameters. Shown are representative OCR traces and the average values of basal respiration (subtracting the OCR after Rot/AA injection from baseline cellular OCR) and maximal respiratory capacity (the difference between FCCP‐ and Rot/AA‐responsive OCR). In the treatment groups, cells were pre‐treated with 0.1 mM DHB or 10 μM UQ_10_ for 5 days prior to the Seahorse XF24 assay. (**A**) Comparison of the OCR of the L111P skin fibroblasts with that of a wild‐type control. (**B**) The effect of DHB treatment on the respiration rate of the L111P skin fibroblasts. (**C**) The effect of supplementation with UQ_10_ on the respiration rate of the L111P skin fibroblasts. Shown on the left are representative OCR traces. Quantitative data are graphed on the right as mean ± S.D. (*n* = 5–10/group). ***P < 0.001 by two‐way anova with Sidak's multiple comparison test. [Colour figure can be viewed at wileyonlinelibrary.com]

We obtained skin fibroblast cells from the L111P patient, in which we observed a ~ 30% decrease in UQ_10_ level and, as expected, accumulation of DMQ_10_ (Fig. 3A and B). Despite the lower UQ content, no significant reduction in OCR was observed in these cells, and they showed no loss of viability in glucose‐free, galactose‐containing medium which forces the cells to predominantly depend on mitochondria for ATP production (Fig. 4A and Fig. S3). Interestingly, in comparison with the fibroblasts from a healthy adult donor which we used as a wild‐type control, the patient's cells showed a slightly higher mitochondrial respiratory capacity (Fig. 4A). This effect might not be related to UQ and could be a result of other differences between the patient's cells and the control used, for example in their genetic background and how they were derived. In fact, under standard culture condition, the patient's skin fibroblasts grew faster than the control. We also observed that the relative amounts of ATP and total ROS were at higher levels in the patient's cells (Fig. S4, Data S1). Alternatively, it is also possible that the higher respiratory capacity in the patient's cells may reflect changes in the RC, for example, increased abundance of RC supercomplexes, which could help to sustain a normal basal rate but also allow for the mitochondria to respire more efficiently when uncoupled. In any case, these findings suggest that neither a moderate decrease of UQ levels nor the presence of the m.1555A>G mutation in the L111P patient has a significantly injurious effect on mitochondrial respiratory function, at least *in vitro*.

We next looked to see whether treatment with DHB could ameliorate the UQ deficiency in the patient's cells. For this, DHB was added into culture media for 1 week at three different doses ranging from 0.1 to 1.5 mM. None of the doses was found to significantly increase cellular UQ_10_ content (Fig. 3A and C). However, all of them completely abolished the accumulation of DMQ_10_ (Fig. 3A and C), indicating that the treatments did indeed impact endogenous UQ biosynthesis. The reason for why there was no significant effect on total UQ content is likely because UQ production from the alternative precursor DHB is offset by inhibition of the native, only partially deficient, UQ biosynthetic pathway 5. In other words, high dose of DHB allows for the COQ7‐bypassing pathway to make more UQ but at the same time inhibits UQ production from the residual natural UQ biosynthetic pathway. The treatment with DHB, however, produced a small but significant increase in the maximal mitochondrial uncoupled respiration rate in the patient's cells (Fig. 4B). Possibly, DMQ, which is very similar to UQ but a poor electron transporter, can compete with UQ for access to RC binding sites, as has been suggested 47. Inhibition of DMQ accumulation could therefore stimulate respiration. A slightly elevated maximal respiration rate was also observed in UQ_10_‐treated patient cells (Fig. 4C).

### UQ and respiratory deficit restoration by DHB in V141E *COQ7* cells

**Figure 5:**
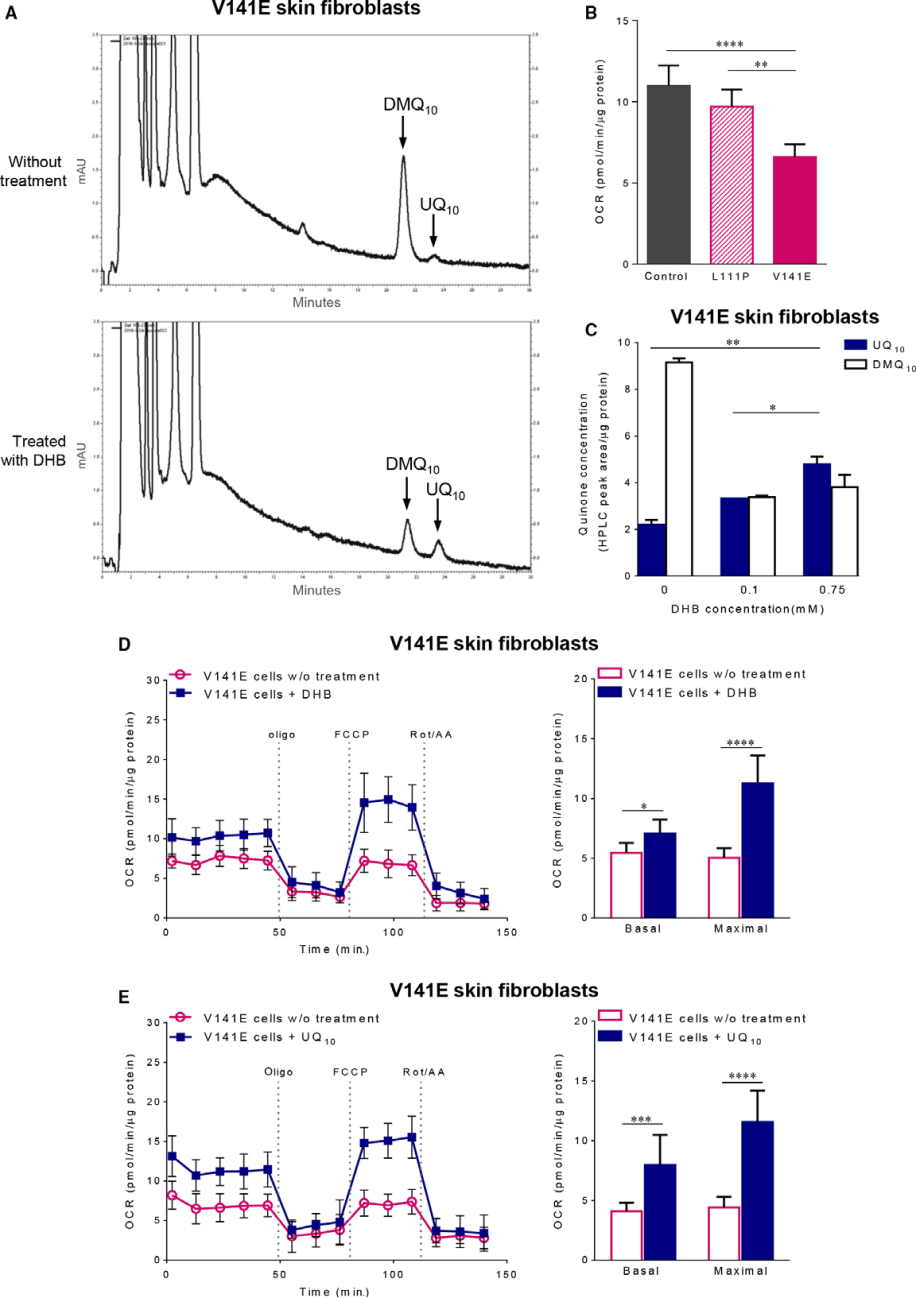
The effects of DHB on the skin fibroblasts derived from the V141E *COQ7* patient. (**A**) HPLC chromatograms of quinone extracts from V141E skin fibroblasts. The patient cells showed a substantial reduction in UQ_10_ levels and a large accumulation of DMQ_10_. After treatment with 0.75 mM DHB, there was a significant increase in UQ_10_ levels, which was accompanied with a lesser accumulation of DMQ_10_. Cells with the same amount of protein (2.1 mg) were used for quinone extraction and HPLC quantitation. (**B**) Basal mitochondrial respiration rates measured using a Seahorse XF24 Analyzer. Data were collected from five cultures in each group and are expressed as means ± S.D. **P < 0.01, ****P < 0.0001 (one‐way anova followed by Tukey's multiple comparison test). (**C**) DHB increases UQ_10_ production and decreases DMQ_10_ accumulation in the V141E patient cells. Quinone levels are expressed as HPLC‐UV peak area normalized to protein content, and shown as mean ± S.E.M. (*n* = 2). *P < 0.05, **P < 0.01 (one‐way anova followed by Tukey's multiple comparison test). (**D**) The effect of DHB treatment on mitochondrial respiration in V141E skin fibroblasts. (**E**) The effect of UQ_10_ on mitochondrial respiration in V141E skin fibroblasts. The experimental condition and data acquisition in **D** and **E** are the same as in Figure 4. Shown on the left are representative OCR traces. Quantitative data are graphed on the right as mean ± S.D. (*n* = 9–10/group). *P < 0.05, ***P < 0.001, ****P < 0.0001 by two‐way anova with Sidak's multiple comparison test.

Fibroblasts of the previously reported *COQ7* patient (V141E) were shown to exhibit severe UQ deficiency 19. However, the previous study did not report DMQ levels, which is a more sensitive and a more direct indication of reduced COQ7 enzymatic activity. Skin fibroblasts derived from the V141E patient were kindly provided by the authors of the study and we sought to determine DMQ_10_ levels. Indeed, large amounts of DMQ_10_ were detected in these cells and they exhibited a significantly greater loss of UQ_10_ than the cells from the L111P patient (Figs 3B, 5A and C). As a result, mitochondrial respiration rate was much lower in these cells than in the L111P patient's cells (Fig. 5B). Despite this, the V141E patient's cells were also capable of surviving in galactose media for at least several days (Fig. S3B). Next we treated the V141E patient's cells with DHB and UQ_10_. In contrast to the L111P patient's cells, both treatments led to a marked increase in both basal and maximal mitochondrial respiratory rates of the V141E cells (Fig. 5D and E). Moreover, DHB increased UQ_10_ levels in a dose‐dependent manner and partially inhibited the production of DMQ_10_. The dose dependency suggests that under the DHB treatment conditions that were used, the V141E patient's cells generated most of their UQ_10_ from the COQ7‐bypassing pathway. A partial inhibition of DMQ_10_ accumulation most likely can be explained by the fact that the blockage of the native UQ biosynthetic pathway caused by loss of COQ7 is much more severe in the V141 patient's cells than in the L111P patient's cells and, therefore, the same level of inhibition by DHB on the native pathway would abolish DMQ completely in the L111P cells but not in the V141E cells.

### Dose‐dependent effects of DHB treatment on *Mclk1* KO animals

**Figure 6:**
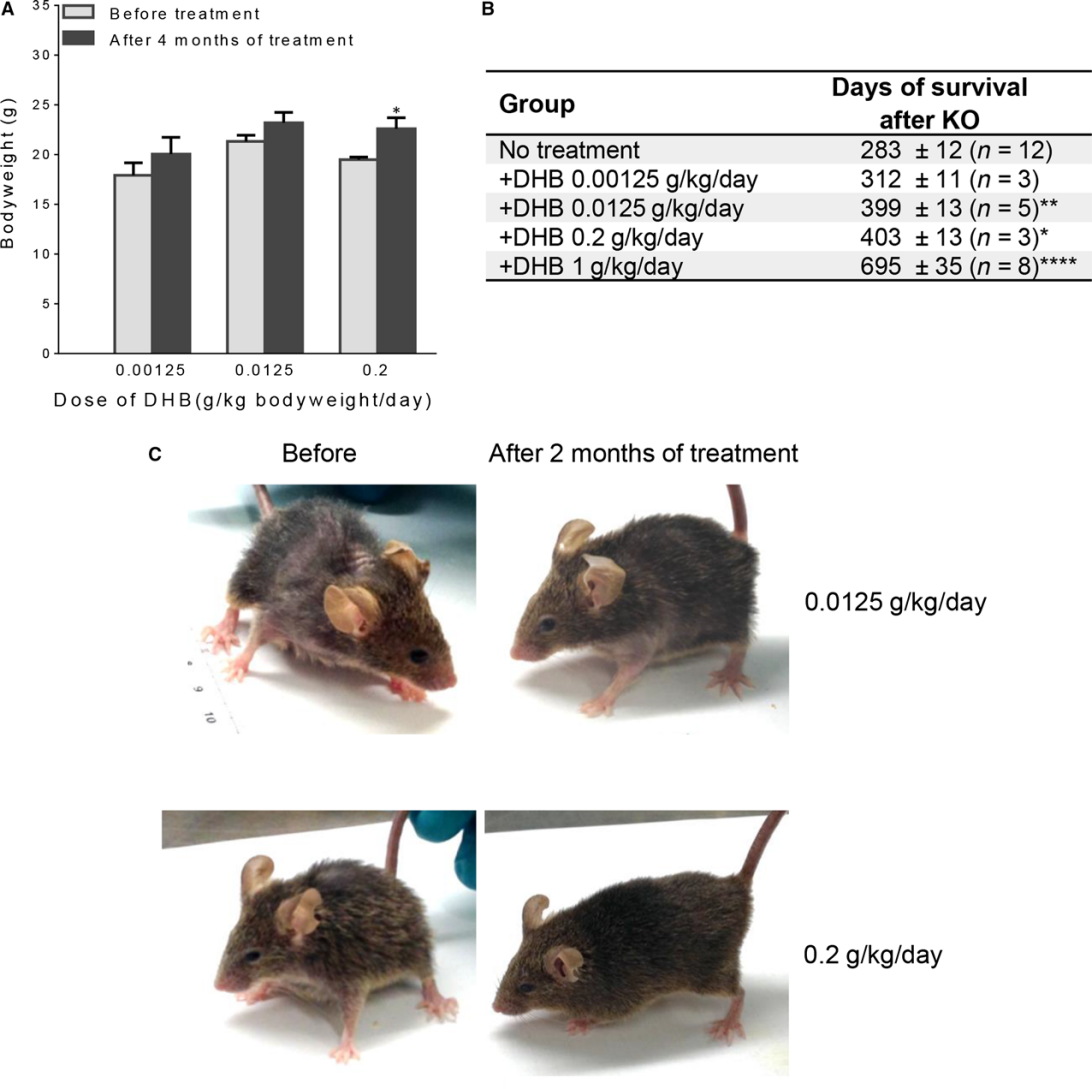
Effects of low‐dose DHB treatment on *Mclk1* KO mice. Whole‐body *Mclk1* KO mice, generated by inducible Cre/loxP‐mediated gene excision 21, were given water containing different concentrations of DHB starting ~ 6 months after completion of KO induction. (**A**) Bodyweight of *Mclk1* KO mice after 4 months of treatment with DHB. Error bars represent S.E.M. *n* = 3–5. *P < 0.05 *versus* bodyweight before start of treatment (Student's *t*‐test). (**B**) Days of survival after completion of KO induction. Data shown are mean survival values ± S.E.M. ***P < 0.0001, ****P < 0.00001 *versus* no treatment group (one‐way anova followed by Dunnett's test). (**C**) Pictures taken before and after 2 months of DHB treatment at the indicated doses.

Administrating DHB to adult‐onset global *Mclk1* KO mice (aog*Mclk1* KO) was shown to dramatically rescue their mutant phenotypes and even allowed them to live a normal lifespan 5. The dose used for this in our previous study was 1 g per kg of bodyweight per day, which would be difficult to achieve in people. Therefore, we sought to find out, using the same mouse model, what the minimum possible dosage required for effective rescue could be. For this, we treated aog*Mclk1* KO mice with DHB at several lower doses, ranging from 0.00125 to 0.2 g/kg/day, starting ~ 6 months after KO induction (but prior to any lethality). Although the lowest dose showed only a minimal effect on bodyweight and survival, at 0.0125 g/kg/day and above there were significant increases in survival, compared with untreated KO animals (Fig. 6).

## Discussion

### Distinct clinical features of the two *COQ7* patients

Compared with the patient reported previously 19, the patient described in this study exhibits fewer and milder symptoms. We speculate that this is most likely because of lesser severity of the patient's UQ deficiency, based on the comparison of quinone levels in skin fibroblasts derived from the patients as well as *in vitro* assessment of the functional impact of the patients' mutations (Figs 2A, 3B, and 5A). The primary effect of UQ deficiency is to impair mitochondrial respiration. However, the exact relationships between UQ levels, mitochondrial function and tissue‐specific effect on cellular health have not been fully established. Recent studies start to show that they are not linear. In cardiomyocytes, severely depressed mitochondrial function due to UQ depletion does not acutely impair organ function 5. And, at least in some cell types (*e.g*. hepatocytes and renal cells), UQ is produced in excess with respect to mitochondrial respiratory activity 5, 46. In the fibroblasts from the L111P patient first reported here, we observed a moderate decrease in UQ levels but no evidence of mitochondrial dysfunction was found (Figs 3B and 4A), which is in line with this scenario.

In addition to the COQ7 T103M polymorphism and L111P mutation which together account for the about 30% decrease in UQ levels, our patient carries a homoplasmic 1555A>G mutation in mitochondrial DNA (OMIM 561000) (Fig. 1B and C). The m.1555A>G mutation, which lies in the *MT‐RNR1* gene, which encodes the mitochondrial ribosome 12S RNA (12S rRNA), is thought to be a risk factor for non‐syndromic hearing loss 27, 38. In particular, it is proposed to predispose carriers to aminoglycoside (AG) ototoxicity which is typically bilateral, irreversible and affecting high frequencies first 38, 48. In addition, it has been reported to be linked to non‐syndromic deafness in the absence of AG exposure, in which case the hearing loss is usually mild and presents at a later time in life (with a ~ 40% probability by the age of 30 years) 38, 49, 50. The patient under study was diagnosed with bilateral sensorineural hearing loss (low and normal frequency) at 5 years of age, although she had not been treated with any AG antibiotics. There are studies indicating that m.1555A>G in itself could impair mitochondrial protein translation, which in turn may cause deficient respiration and participate in the pathogenicity of m.1555A>G on hearing 51, 52. Inner ear hair cells and neurons are particularly dependent upon mitochondria to meet their high energy demands, which potentially explains a high prevalence of hearing loss in mitochondrial diseases 53. Possibly, in the patient's hair cells (in contrast to the slowly respiring fibroblasts), UQ deficiency and the 1555A>G mitochondrial mutation lead to a synergistic inhibition of mitochondrial function beyond a threshold, causing irreversible damages and/or cell death and finally the clinical manifestation of hearing loss.

Another main symptom of the patient is spasticity. Skeletal muscles are often affected in mitochondrial diseases including due to UQ deficiency 54, 55. However, the patient's muscle biopsy did not show any evidence of abnormal mitochondrial pathology. Thus, it is quite possible that spasticity in her lower limbs is not caused by a mitochondrial defect in skeletal muscles; rather, it could be the result of peripheral polyneuropathy. In fact, the CSF lactate level was found to be elevated in the patient, indicating possible mitochondrial cytopathy affecting the nervous system. Patients with mitochondrial disorders often develop peripheral neuropathy, although it is rare to have that as a predominant clinical presentation 56. Peripheral nerves have long axons; therefore, they are believed to be metabolically vulnerable, likely explaining their frequent involvement in mitochondrial diseases. Of the UQ deficiency patients reported so far, mutations in the *ADCK3* (*COQ8*) or *PDSS1* gene were reported to experience peripheral or axonal neuropathy, in addition to other symptoms 10, 13. In the V141E *COQ7* patient, electrophysiological abnormalities were detected at 2 years of age, suggesting peripheral sensorimotor polyneuropathy of axonal and demyelinating type 19. More interestingly, a variable degree of spasticity was reported in *ADCK3* patients 57. Thus, it is possible that in the L111P patient, UQ deficiency in nerve cells is manifested in peripheral motor neuropathy leading to spasticity.

### Different responses to DHB treatment of different COQ7 mutant cells

We showed that the two mutations, L111P and V141E, both dramatically reduce COQ7 protein levels (Fig. 2B). However, the V141E mutation is more severe by most measures including its effect on quinone content and mitochondrial RC function (Fig. 5A and B). We confirmed that DHB increased UQ levels in skin fibroblasts from the V141E patient and showed that this, in turn, alleviates impaired mitochondrial respiration (Fig. 5C and D). In contrast, the cells carrying the mild mutation, L111P, showed no improvement in coupled mitochondrial respiration in response to DHB treatment (Fig. 4B). Most likely, the differences in the responses to DHB reflect differences in residual COQ7 activity. In the cells that were supplemented with DHB, the rate of UQ synthesis is the aggregate of DHB‐dependent, COQ7‐independent, synthesis and of UQ synthesis from the native, COQ7‐dependent, pathway, with the latter pathway being partially inhibited by DHB. Thus, cells that have a more complete loss of COQ7 are more likely to benefit from DHB, because, if native UQ biosynthesis is very low, its inhibition by DHB will have a smaller effect on net UQ yield. Indeed, the dose‐dependent UQ increase seen in the DHB‐treated V141E cells suggests that UQ is mainly synthesized from DHB in the DHB‐treated patient cells (Fig. 5C), as was previously described for *Mclk1* KO mouse mutants 5. By contrast, treatment of the L111P cells with DHB had no effect on total UQ levels but nevertheless was sufficient to inhibit all DMQ accumulation (Fig. 3C). Presumably, the balance between alternative synthesis and inhibition of the natural pathway resulted in UQ levels very similar to what is found in untreated cells. In line with this, it is of note that DHB treatment was also shown to have different effects on two different mouse models carrying a homozygous mutation in the *Coq9* gene 46. *Coq9* mutations produce deficiency of UQ by reducing the expression level of MCLK1/COQ7 22, 46. The study showed that only in the more severe model, in which residual UQ were about 20% of the wild‐type level, could DHB treatment significantly increase UQ tissue levels 46.

### The challenges of treating COQ7 deficiency patients with DHB

Our findings and the previous report by Freyer *et al*. 19 strongly suggest that the V141E COQ7 patient could benefit from DHB as well as, possibly, supplementation of exogenous UQ_10_. In contrast, both treatments are unlikely to be helpful to the L111P patient. For *in vivo* DHB treatment, our study with *Mclk1* KO mice suggests that the minimal effective dosage given orally in the drinking water is 0.0125 g/kg/day (Fig. 6), which is still difficult to achieve in people considering the treatment needs to be continuous for a lifetime. However, a lower but effective dosage could be possible if better formulations and more efficient drug delivery systems are used than what we employed for mice. Lastly, it is worth noting that the efficacy of DHB treatment could be tissue specific, given that the efficacy of DHB treatment depends on the extent of the loss of COQ7 enzymatic activity. Indeed, the amount of residual COQ7 protein likely depends on cell type‐specific characteristics, such as cell renewal and protein turnover. Despite these caveats, the possibility of treatment of the most severe *COQ7* patients with DHB should be studied.

## Conflict of interest

There are no competing interests related to the current study.

## Supporting information


**Figure S1** UQ levels in the mouse *Mclk1/Coq7* knockout (KO) mouse embryonic fibroblasts re‐expressing wild‐type or mutant *Mclk1*.
**Figure S2** Verification of the identities of UQ10 and DMQ10 HPLC‐UV chromatographic peaks.
**Figure S3** The skin fibroblasts derived from *COQ7* patients are viable in galactose media.
**Figure S4** ATP level and relative oxygen species (ROS) production in L111P skin fibroblasts.
**Figure S5** Original uncropped scans of western blots displayed in Figure 2.
**Data S1** Supplemenatary methods.
**Data S2** Supplementary notes.
