# Supplementary material for: Pathogenicity of two COQ7 mutations and responses to 2,4‐dihydroxybenzoate bypass treatment
Source: J Cell Mol Med. 2017 Apr 13;21(10):2329–43. doi: 10.1111/jcmm.13154 (PMC5618687; doi:10.1111/jcmm.13154)
Supplement: Supplementary file 1 — Figure S1 UQ levels in the mouse Mclk1/Coq7 knockout (KO) mouse embryonic fibroblasts re‐expressing wild‐type or mutant Mclk1. Figure S2 Verification of the identities of UQ10 and DMQ10 HPLC‐UV chromatographic peaks. Figure S3 The skin fibroblasts derived from COQ7 patients are viable in galactose media. Figure S4 ATP level and relative oxygen species (ROS) production in L111P skin fibroblasts. Figure S5 Original uncropped scans of western blots displayed in Figure 2. Data S1 Supplemenatary methods. Data S2 Supplementary notes. [file JCMM-21-2329-s001.pdf]

Supporting information

It includes 5 Supplementary Figures, Supplementary Methods, and a Supplementary Note.

SUPPLEMENTARY FIGURES

A

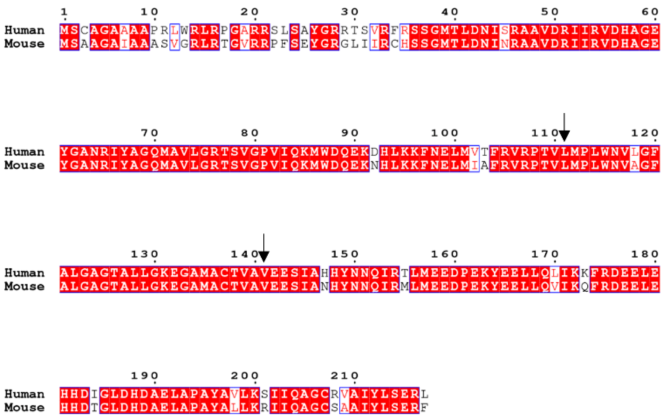

B

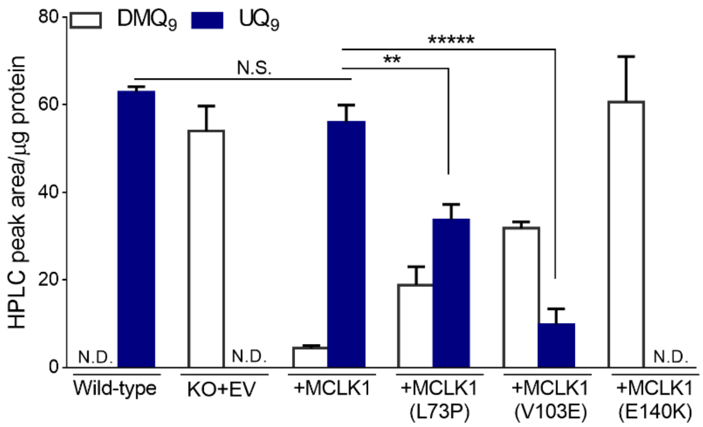

Figure S1. UQ levels in the mouse *Mclk1/Coq7* knockout (KO) mouse embryonic fibroblasts re-expressing wild-type or mutant *Mclk1*. (A) Sequence alignment of human and mouse COQ7. Alignment of protein sequences is exhibited using ESPript 3.0. Conserved residues are indicated in red. Arrows indicate the positions of the mutations (L111P and V141E) described in this study. The L111 and V141 amino acids are conserved between humans and mice. **(B)** UQ levels in the *Mclk1* KO mouse embryonic fibroblasts re-expressing wild-type or mutant *Mclk1*. c.217T>C&c.218T>C[p.Leu73Pro] (L73P) and c.308T>A[p.Val103Glu] (V103E) in the mouse MCLK1 correspond to the patient mutations L111P and V141E, respectively. Both of them decreased UQ_9 levels and caused accumulation of DMQ_9. The effect of V103E was more severe than that of L73P. The c. 418G>A[p.Glu140Lys] (E140K) change, which corresponds to the *C. elegans* mutant *clk-1(2519)*, resulted in a complete loss of protein function as no UQ was detected in the KO cells expressing this mutant form. EV: empty vector. N.D.: Not detectable. N.S.: not significant. Quinone levels are expressed as HPLC-UV peak area normalized to protein content. Error bars represent SEM (n=4-6). $^{**}p<0.01$, $^{****}p<0.0001$ (one-way ANOVA followed by Tukey's post hoc test).

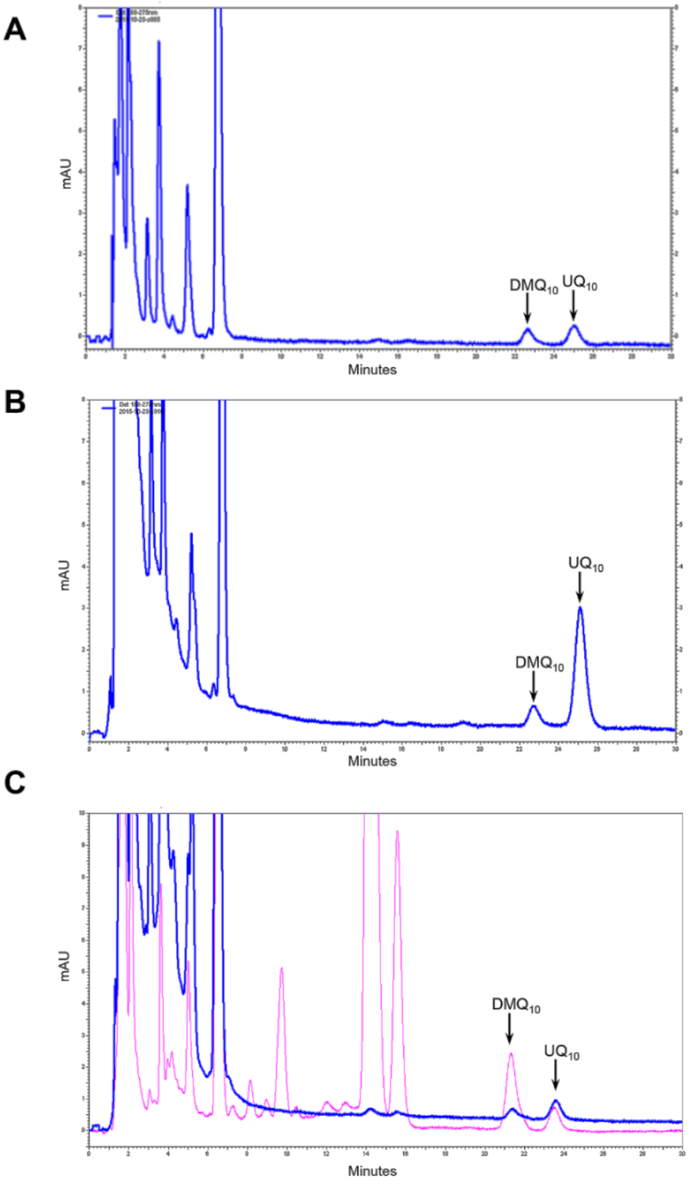

Figure S2. Verification of the identities of UQ_{10} and DMQ_{10} HPLC-UV chromatographic peaks. (A) A representative HPLC-UV chromatogram of quinone extract from L111P skin fibroblasts. The identity of quinone peaks was determined based on their retention time. **(B)** Spiking the quinone extract from L111P patient cells with UQ_{10} standard increased the height of the peak corresponding to the migration time of UQ_{10}. **(C)** Overlay of HPLC-UV chromatogram of a quinone extract from L111P patient cells (blue) with that from a mouse kidney lacking the expression of MCLK1/COQ7 and thus accumulating DMQ (pink). **B, C** further confirm the initial identification of DMQ_{10} and UQ_{10} analyte peaks.

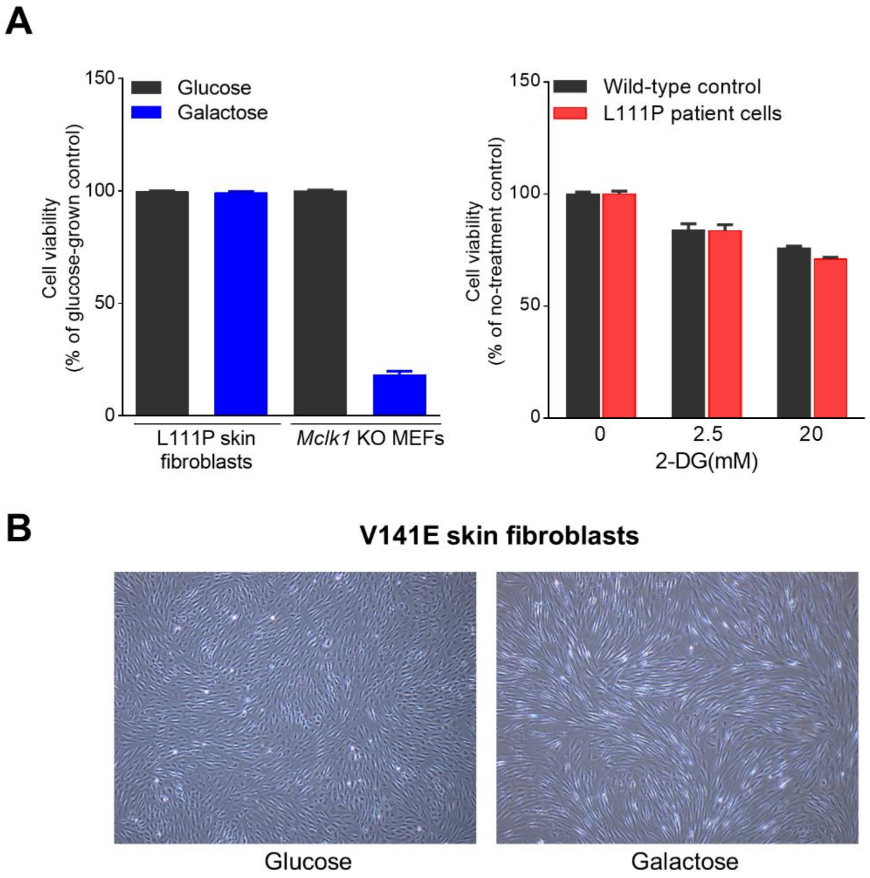

Figure S3. The skin fibroblasts derived from *COQ7* patients are viable in galactose media. (A) L111P skin fibroblasts showed neither decreased viability in glucose-free galactose-containing media nor increased sensitivity to the glucose inhibitor 2-deoxyglucose (2-DG). Cell viability was measured using AlamarBlue. *Mclk1* KO mouse embryonic fibroblasts (MEFs) were used as a positive control as they are not viable in galactose media. Data shown are the mean ± SEM of 4 replicate wells. **(B)** The skin fibroblasts derived from the V141E patient grew slowly but were viable in galactose media. Phase contrast cell images were taken after 5 days' culture in glucose-free galactose-containing culture media.

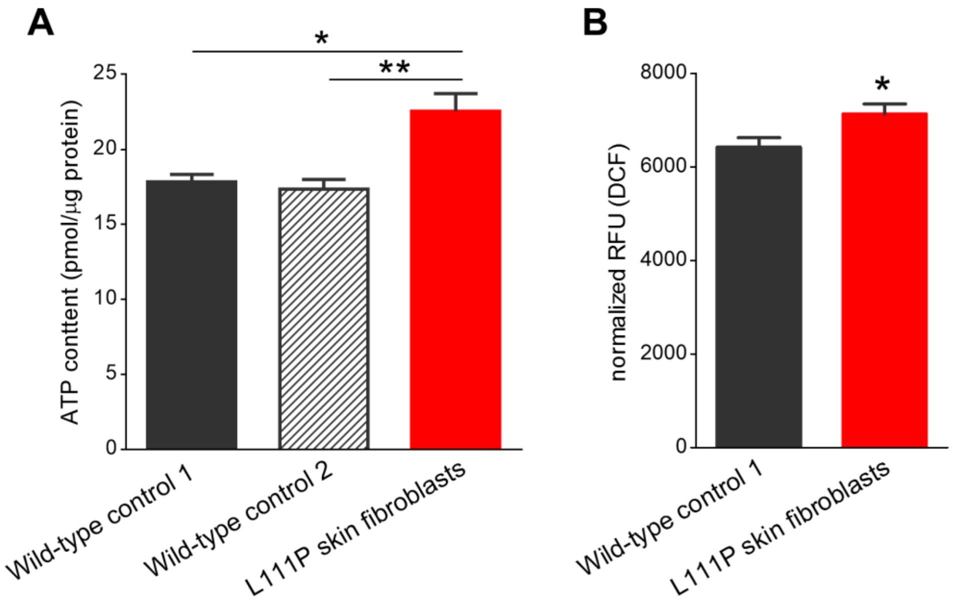

Figure S4. ATP level and relative oxygen species (ROS) production in L111P skin fibroblasts. (A) ATP levels normalized to total cellular protein. Data represent mean ± SEM. n=4. *$p<0.05$, **$p<0.01$ (One-way ANOVA followed by Tukey's multiple comparison test). **(B)** ROS-dependent dichlorofluorescein (DCF) fluorescence. Relative fluorescence intensities (RFU, at 485/530 nm) of DCF were normalized to total cellular protein content. Error bars are SEM. n=12. *$p<0.05$ (unpaired Student's *t*-test). Wild-type control line 1 were fibroblasts derived from skin biopsy obtained from a 30 years old healthy donor. Wild-type control line 2 were fibroblasts derived from skin biopsy obtained from a healthy newborn baby.

Figure 2B

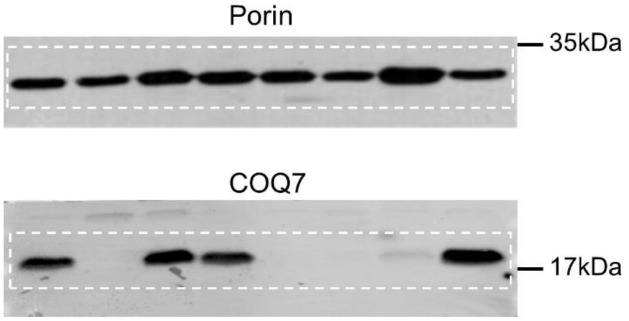

Figure 2C

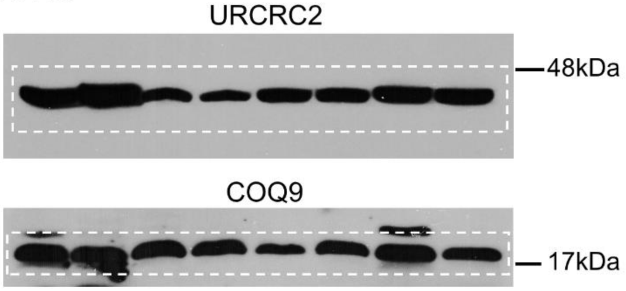

Figure S5. **Original uncropped scans of western blots displayed in Figure 2.** Dashed boxes indicate regions that were cropped. Positions of protein size markers are shown on the right.

SUPPLEMENTARY METHODS

Measurement of reactive oxygen species (ROS)
Intracellular ROS were estimated using a fluorescent probe, 6-carboxy-2’,7’-dicholorfluorescin diacetate (DCFH-DA). Cells were seeded in black 96-well plates and washed once with PBS before incubation with the probe. After the cells were loaded with DCFH-DA (10 μM) and incubated at 37°C for 30 min in the dark, the fluorescence intensities were measured at an excitation wavelength of 485 nm and an emission wavelength of 530 nm using a TECAN plate reader. Relative fluorescence intensities were normalized by the protein amount in each well (determined by the Thermo Scientific Pierce BCA Protein Assay Kit).

Cellular ATP determination
Exponentially growing patient and control fibroblasts were lysed on ice in buffer containing 5 mM Tris-HCl (pH 7.75) and 4 mM EDTA. The cell lysates were then boiled for 5 minutes at 95°C and the clear supernatants were used to determine ATP content using a luciferin/luciferase bioluminescent assay (Promega) following the manufacturer's instructions.

SUPPLEMENTARY NOTE

This work was partially funded by the Canadian Rare Diseases Models and Mechanisms (RDMM) Network’s catalyst grant. Listed below are the current members of the scientific and clinical advisory committees of RDMM.

Scientific advisory committee: **Co-chairs:** Philip Hieter, Richard Rachubinski, **Members:** Berman, Boycott, Campeau, Hicks, Junker, Leroux, Lipshitz, Masson, McMaster, Rossant, Shoubridge. Clinical advisory committee: Co-chairs: Kym Boycott, Philippe Campeau Members: Cohn, Hamilton, Junker, MacDonald, Turvey, van Karnebeek, Michaud.
